# Supplementary material for: Climatic change and extinction risk of two globally threatened Ethiopian endemic bird species
Source: PLoS One. 2021 May 19;16(5):e0249633. doi: 10.1371/journal.pone.0249633 (PMC8133463; doi:10.1371/journal.pone.0249633)
Supplement: S1 File — (DOCX) [file pone.0249633.s001.docx]

**Climate change and extinction risk of two globally threatened Ethiopian endemic bird species**

Andrew J. Bladon^1*^, Paul F. Donald^1,2,3^, Nigel J. Collar^2^, Jarso Denge^4^, Galgalo Dadacha^4^, Mengistu Wondafrash^5^ and Rhys E. Green^1,3^.

^1^ Conservation Science Group, Department of Zoology, The David Attenborough Building, University of Cambridge, Pembroke Street, Cambridge CB2 3QZ, UK.

^2^ BirdLife International, The David Attenborough Building, University of Cambridge, Pembroke Street, Cambridge CB2 3QZ, UK.

^3^ RSPB Centre for Conservation Science, RSPB, The Lodge, Sandy, Bedfordshire SG19 2DL, UK.

^4^ Borana National Park Authority, PO Box 34, Yabello, Borana Zone, Oromiya, Ethiopia.

^5^ Ethiopian Wildlife and Natural History Society, PO Box 13303, Bole Sub City, Addis Ababa, Ethiopia.

* Corresponding author

Email: [andrew.j.bladon@gmail.com](mailto:andrew.j.bladon@gmail.com) (AJB)

**S1 Table:** Six Global Circulation Models (GCMs) used for the projection of bioclimate variables under future climate scenarios.

| **Global Circulation Model (GCM)** | **Developers** | **Reference** |
| --- | --- | --- |
| BCC-CSM1-1 | Beijing Climate Center, Beijing, China | (Wu et al. 2013) |
| CCSM4 | National Center for Atmospheric Research, Boulder, Colorado, USA | (Meehl et al. 2012) |
| HadGEM2-ES | Hadley Centre for Climate Science and Services, Exeter, UK | (Collins et al. 2008) |
| IPSL-CM5A-LR | Institut Pierre Simon Laplace, France | (Dufresne et al. 2013) |
| MIROC-ESM | Center for Climate System Research, Tokyo, Japan | (Hasumi and Emori 2004) |
| MRI-CGCM3 | Meteorological Research Institute, Japan | (Yukimoto et al. 2012) |

**S2 Table:** Current and projected future values of five bioclimate variables within a convex hull fitted around the current distributions of the White-tailed Swallow and the Ethiopian Bush-crow. Current values are the minimum, mean and maximum values across all cells within the species’ range. Projected values are the minimum, mean and maximum values of the mean across all cells within the species’ current range under six different Global Circulation Models (Table S1) at each of two years (2050 and 2070) and under each of four Representative Concentration Pathway (RCP) scenarios (IPCC 2014). Bioclimate variables are abbreviated as follows: Precip wet = precipitation of the wettest quarter; Precip dry = precipitation of the driest quarter; Temp season = Temperature seasonality; Max temp = Maximum temperature of the warmest month; Temp range = Annual temperature range (Hijmans et al. 2005). The current range used for the swallow includes two hulls fitted around the core range and the Liben Plain independently, but does not include the area in between.

|  | | | **White-tailed Swallow** | | | **Ethiopian Bush-crow** | | |
| --- | --- | --- | --- | --- | --- | --- | --- | --- |
| **Year** | **RCP** | **Bioclimate Variable** | **Min.** | **Mean** | **Max.** | **Min.** | **Mean** | **Max.** |
| Current | - | Precip wet (mm) | 196.0 | 305.1 | 433.7 | 241.9 | 307.8 | 385.3 |
| Current | - | Precip dry (mm) | 15.0 | 27.6 | 92.4 | 19.0 | 28.5 | 92.4 |
| Current | - | Temp season | 853.8 | 1308.4 | 1712.4 | 954.2 | 1269.2 | 1563.6 |
| Current | - | Max temp (°C) | 24.2 | 30.0 | 36.4 | 25.6 | 29.1 | 32.3 |
| Current | - | Temp range (°C) | 15.2 | 18.0 | 22.9 | 15.9 | 17.7 | 20.5 |
| 2050 | 2.6 | Precip wet (mm) | 282.3 | 427.2 | 823.1 | 283.1 | 430.4 | 833.4 |
| 2050 | 2.6 | Precip dry (mm) | 26.5 | 32.1 | 43.8 | 27.2 | 33.2 | 45.7 |
| 2050 | 2.6 | Temp season | 974.9 | 1272.4 | 1594.2 | 940.8 | 1230.7 | 1546.6 |
| 2050 | 2.6 | Max temp (°C) | 30.1 | 30.8 | 31.3 | 29.1 | 29.9 | 30.4 |
| 2050 | 2.6 | Temp range (°C) | 16.5 | 17.3 | 18.0 | 16.3 | 17.0 | 17.7 |
| 2070 | 2.6 | Precip wet (mm) | 319.3 | 429.6 | 661.0 | 322.1 | 434.5 | 672.1 |
| 2070 | 2.6 | Precip dry (mm) | 23.8 | 28.8 | 35.0 | 24.4 | 29.7 | 36.4 |
| 2070 | 2.6 | Temp season | 936.8 | 1291.1 | 1544.9 | 903.4 | 1251.3 | 1503.2 |
| 2070 | 2.6 | Max temp (°C) | 30.0 | 30.9 | 31.4 | 29.0 | 29.9 | 30.5 |
| 2070 | 2.6 | Temp range (°C) | 16.5 | 17.4 | 18.1 | 16.2 | 17.1 | 17.8 |
| 2050 | 4.5 | Precip wet (mm) | 257.1 | 434.6 | 960.5 | 258.3 | 440.3 | 973.8 |
| 2050 | 4.5 | Precip dry (mm) | 16.2 | 27.0 | 41.9 | 16.9 | 28.1 | 43.8 |
| 2050 | 4.5 | Temp season | 1133.9 | 1333.4 | 1578.9 | 1093.5 | 1290.3 | 1530.8 |
| 2050 | 4.5 | Max temp (°C) | 30.9 | 31.5 | 32.1 | 29.9 | 30.5 | 31.1 |
| 2050 | 4.5 | Temp range (°C) | 15.7 | 17.3 | 17.9 | 15.3 | 17.0 | 17.6 |
| 2070 | 4.5 | Precip wet (mm) | 306.9 | 504.3 | 1058.3 | 312.2 | 513.1 | 1075.8 |
| 2070 | 4.5 | Precip dry (mm) | 15.0 | 26.6 | 40.7 | 15.7 | 27.7 | 42.8 |
| 2070 | 4.5 | Temp season | 1065.5 | 1358.8 | 1580.6 | 1024.9 | 1315.4 | 1532.7 |
| 2070 | 4.5 | Max temp (°C) | 31.6 | 31.9 | 32.6 | 30.6 | 30.9 | 31.6 |
| 2070 | 4.5 | Temp range (°C) | 15.6 | 17.3 | 18.1 | 15.2 | 17.0 | 17.8 |
| 2050 | 6.0 | Precip wet (mm) | 294.9 | 404.3 | 621.1 | 297.4 | 408.2 | 626.5 |
| 2050 | 6.0 | Precip dry (mm) | 17.7 | 28.2 | 44.5 | 18.9 | 29.2 | 46.3 |
| 2050 | 6.0 | Temp season | 1208.9 | 1362.6 | 1507.4 | 1168.3 | 1319.1 | 1462.8 |
| 2050 | 6.0 | Max temp (°C) | 31.2 | 31.7 | 32.1 | 30.2 | 30.7 | 31.1 |
| 2050 | 6.0 | Temp range (°C) | 16.9 | 17.7 | 18.1 | 16.5 | 17.4 | 17.8 |
| 2070 | 6.0 | Precip wet (mm) | 321.0 | 457.8 | 925.3 | 322.1 | 463.0 | 933.4 |
| 2070 | 6.0 | Precip dry (mm) | 15.9 | 27.7 | 48.8 | 16.7 | 28.7 | 51.7 |
| 2070 | 6.0 | Temp season | 1165.2 | 1324.5 | 1556.0 | 1113.5 | 1280.5 | 1509.3 |
| 2070 | 6.0 | Max temp (°C) | 30.8 | 31.9 | 32.7 | 29.8 | 30.9 | 31.8 |
| 2070 | 6.0 | Temp range (°C) | 15.7 | 17.3 | 18.0 | 15.3 | 17.0 | 17.8 |
| 2050 | 8.5 | Precip wet (mm) | 301.0 | 521.4 | 1165.5 | 300.3 | 531.0 | 1184.8 |
| 2050 | 8.5 | Precip dry (mm) | 14.4 | 29.9 | 51.8 | 15.1 | 31.1 | 54.8 |
| 2050 | 8.5 | Temp season | 1069.1 | 1364.1 | 1689.7 | 1025.8 | 1318.1 | 1637.9 |
| 2050 | 8.5 | Max temp (°C) | 31.3 | 31.9 | 32.7 | 30.3 | 30.9 | 31.7 |
| 2050 | 8.5 | Temp range (°C) | 15.6 | 17.2 | 18.1 | 15.2 | 16.9 | 17.8 |
| 2070 | 8.5 | Precip wet (mm) | 300.5 | 566.8 | 1537.6 | 301.0 | 576.1 | 1555.7 |
| 2070 | 8.5 | Precip dry (mm) | 19.0 | 32.7 | 62.3 | 20.1 | 33.7 | 65.2 |
| 2070 | 8.5 | Temp season | 1023.9 | 1395.6 | 1739.4 | 985.8 | 1350.3 | 1688.0 |
| 2070 | 8.5 | Max temp (°C) | 32.0 | 32.9 | 34.1 | 31.0 | 31.9 | 33.1 |
| 2070 | 8.5 | Temp range (°C) | 15.6 | 17.2 | 18.1 | 15.1 | 16.9 | 17.9 |

**S3 Table:** Delta AUC scores for models of White-tailed Swallow distribution, fitted without each climate variable in turn, under the three best-performing model algorithms (MaxEnt, GLM, GAM), and compared to values for the best-performing model (a Boosted Regression Tree, BRT) for the Ethiopian Bush-crow, taken from Bladon et al. (2019).

|  | **White-tailed Swallow** | | | **Ethiopian Bush-crow** |
| --- | --- | --- | --- | --- |
| **Variable removed** | **MAXENT** | **GLM** | **GAM** | **BRT** |
| Precipitation of the wettest quarter | 0.011 | -0.014 | 0.038 | 0.001 |
| Precipitation of the driest quarter | 0.146 | 0.047 | 0.089 | 0.018 |
| Temperature seasonality | 0.046 | -0.015 | -0.014 | 0.012 |
| Maximum temperature | 0.121 | 0.040 | 0.028 | 0.022 |
| Annual temperature range | 0.047 | 0.012 | -0.044 | 0.000 |


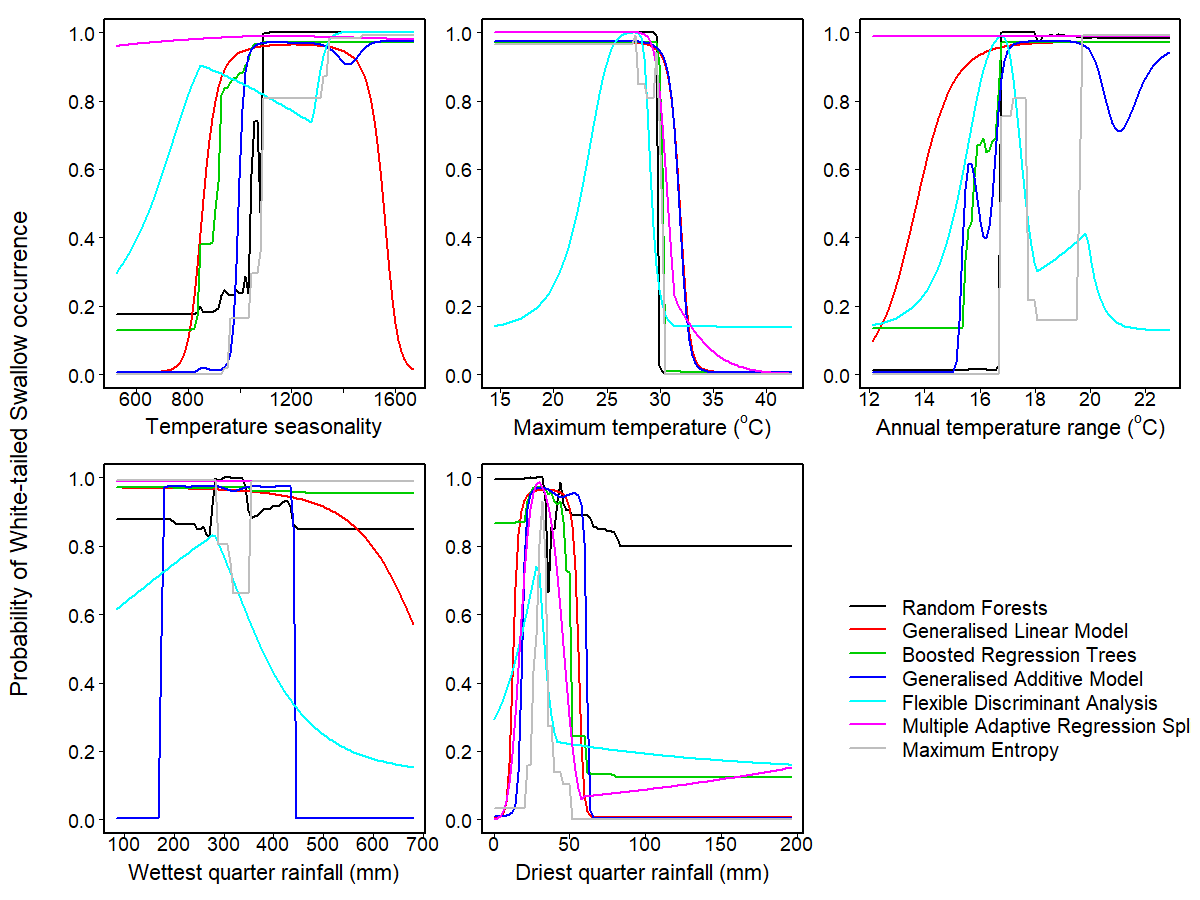


**S1 Fig:** The response of partial probability of White-tailed Swallow occurrence to five bioclimatic variables. Lines show the predicted responses to each variable according to each of seven model algorithms fitted using the ‘biomod2’ package (Thuiller et al. 2012) in R (R Core Team 2019), with all other variables held constant.

**References**

Bladon, A. J., P. F. Donald, S. E. I. Jones, N. J. Collar, J. Deng, G. Dadacha, Y. D. Abebe, and R. E. Green (2019). Behavioural thermoregulation and climatic range restriction in the globally threatened Ethiopian Bush-crow *Zavattariornis stresemanni*. Ibis 161:546–558.

Collins, W. J., N. Bellouin, M. Doutriaux-Boucher, N. Gedney, T. Hinton, C. D. Jones, S. Liddicoat, G. Martin, F. O’Connor, J. Rae, and C. Senior (2008). Evaluation of the HadGEM2 model. Met Office Hadley Centre 74.

Dufresne, J.-L., M.-A. Foujols, S. Denvil, A. Caubel, O. Marti, O. Aumont, Y. Balkanski, S. Bekki, H. Bellenger, R. Benshila, S. Bony, et al. (2013). Climate change projections using the IPSL-CM5 Earth System Model: from CMIP3 to CMIP5. Climate Dynamics 40:2123–2165.

Hasumi, H., and S. Emori (2004). K-1 coupled GCM (MIROC) description. Center for Climate System Research, University of Tokyo.

Hijmans, R. J., S. E. Cameron, J. L. Parra, P. G. Jones, and A. Jarvis (2005). Very high resolution interpolated climate surfaces for global land areas. International Journal of Climatology 25:1965–1978.

Meehl, G. A., W. M. Washington, J. M. Arblaster, A. Hu, H. Teng, C. Tebaldi, B. N. Sanderson, J.-F. Lamarque, A. Conley, W. G. Strand, and J. B. White (2012). Climate System Response to External Forcings and Climate Change Projections in CCSM4. Journal of Climate 25:3661–3683.

R Core Team (2019). R: A language and environment for statistical computing. R Foundation for Statistical Computing, Vienna, Austria.

Thuiller, W., D. Georges, and R. Engler (2012). Package “biomod2” - Ensemble Platform for species distribution modelling.

Wu, T., W. Li, J. Ji, X. Xin, L. Li, Z. Wang, Y. Zhang, J. Li, F. Zhang, M. Wei, X. Shi, et al. (2013). Global carbon budgets simulated by the Beijing Climate Center Climate System Model for the last century. Journal of Geophysical Research: Atmospheres 118:4326–4347.

Yukimoto, S., Y. Adachi, M. Hosaka, T. Sakami, H. Yoshimura, M. Hirabara, T. Y. Tanaka, E. Shindo, H. Tsujino, M. Deushi, R. Mizuta, et al. (2012). A New Global Climate Model of the Meteorological Research Institute: MRI-CGCM3 —Model Description and Basic Performance—. Journal of the Meteorological Society of Japan. Ser. II 90A:23–64.
